# Syndrome of Ventricular Septal Defect and Aortic Regurgitation - A 22-Year Review of its Management

**DOI:** 10.21470/1678-9741-2020-0207

**Published:** 2021

**Authors:** Sivakumar Krishnasamy, Sivakumar Sivalingam, Jeswant Dillon, Raja Amin Raja Mokhtar, A. Yakub, Ramesh Singh

**Affiliations:** 1 Department of Surgery, Cardiothoracic Surgery, University Malaya, Kuala Lumpur, Malaysia.; 2 Department of Cardiothoracic Surgery, National Heart Institute, Kuala Lumpur, Malaysia.; 3 Department of Medicine, Cardiology Division, University Malaya, Kuala Lumpur, Malaysia.

**Keywords:** Aortic Valve Insufficiency, Reoperation, Heart Septal Defects, Ventricular, Heart Valve Prosthesis, Cardiac Surgical Procedures, Prolapse

## Abstract

**Introduction:**

The presence of aortic regurgitation (AR) in the setting of ventricular septal defect (VSD) has always been a management challenge.

**Methods:**

This is a retrospective study looking at patients who underwent VSD closure with or without aortic valve intervention between January 1st, 1992 and December 31st, 2014 at the Institute Jantung Negara. This study looked at all cases of VSD and AR, where AR was classified as mild, moderate, and severe, the intervention done in each of this grade, and the durability of that intervention. The interventions were classified as no intervention (NI), aortic valve repair (AVr), and aortic valve replacement (AVR).

**Results:**

A total of 261 patients were recruited into this study. Based on the various grades of AR, 105 patients had intervention to their aortic valve during VSD closure. The rest 156 had NI. All patients were followed up for a mean time of 13.9±3.5 years. Overall freedom from reoperation at 15 years was 82.6% for AVr. Various factors were investigated to decide on intervening on the aortic valve during VSD closure. Among those that were statistically significant were the grade of AR, size of VSD, age at intervention, and number of cusp prolapse.

**Conclusion:**

We can conclude from our study that all moderate and severe AR with small VSD in older patients with more than one cusp prolapse will need intervention to their aortic valve during the closure of VSD.

**Table:** 

Abbreviations, acronyms & symbols				
AR	= Aortic regurgitation		HR	= Hazard ratio
AVr	= Aortic valve repair		IJN	= Institute Jantung Negara
AVR	= Aortic valve replacement		NI	= No intervention
CI	= Confidence interval		PM	= Perimembranous
DCSA	= Doubly committed subaortic		VSD	= Ventricular septal defect

## INTRODUCTION

Combination of ventricular septal defect (VSD) and aortic regurgitation (AR) due to prolapse of right coronary or, less frequently, non-coronary cusp is known as Laubry-Pezzi syndrome^[1]^. Early VSD closure have been proposed to prevent the onset of AR or the worsening of the existing AR.

However, once an aortic valve deformity is present, surgical closure of VSD alone without intervention to the aortic valve may not be enough to prevent progressive AR. These patients will require aortic valve repair (AVr) or aortic valve replacement (AVR). Aortic valve prolapse and AR are more frequent and severe in patients with delayed surgery, highlighting the importance of early surgical intervention.

In patients with VSD and concomitant AR, moderate and severe AR represent a challenging surgical issue. AVR is often associated with major drawbacks.

Mechanical prosthetics require long-term anticoagulation therapy and are often limited by the size of the aortic annulus. On the other hand, homografts and bioprosthetic valves have a high rate of early calcification and failure. Therefore, AVr has been an attractive alternative in the treatment of AR instead of AVR. Hence, this study is aimed mainly at looking at the long-term outcome of aortic valve intervention done in patients with syndrome of VSD and AR.

### Objectives

### Primary Objective

To assess the outcome of aortic valve intervention in patients with AR in the setting of VSD.

### Secondary Objective

To identify variables that may predict the outcome of AVr and the risk of reoperation in our study population.

## METHODS

This is a retrospective study. Patients with VSD and concomitant AR who underwent VSD repair with or without aortic valve intervention between January 1^st^, 1992 and December 31^st^, 2014 at the Institute Jantung Negara (IJN), Kuala Lumpur (Malaysia), were included in this study. The patients were classified into two major groups where in one group the severity of AR was classified as mild, moderate, and severe, and in the other group the type of aortic valve intervention was classified as no intervention (NI), AVr, and AVR. Correlations between the severity of AR and the type of intervention used to address the AR were all analyzed, and the outcomes of each of these were reviewed together with the factors that contributed to them.

### Sample Selection

### Inclusion Criteria

All patients with VSD and concomitant AR who underwent VSD closure with or without intervention to the aortic valve.

### Exclusion Criteria

- Patients with other major cardiac anomalies.
- Patients with congenital syndromes.
- Patients with other comorbidities who are unfit for VSD closure.
- Patients with connective tissue disease or aortopathy features.

### Data Collection

The operation theatre logbooks were reviewed from January 1^st^, 1992 up to December 31^st^, 2014. Ethics approval to review all the patients’ medical records were obtained for the Ethics Committee of IJN, Kuala Lumpur, in 2012. All patients who underwent VSD closure were identified and their medical records were reviewed. Only those who had concomitant AR were included in this study.

Patients were divided into three groups - mild AR, moderate AR, and severe AR - based on their preoperative echocardiographic findings. Further information regarding aortic valve intervention for AR was obtained from the operative notes. They were further subdivided into three groups based on the intervention performed - AVr, AVR, and NI.

Postoperative echocardiographic results were reviewed from patients’ case notes to assess improvement in the grade of AR after intervention (prior to discharge). All the echocardiographic assessment was done transthoracically as, until 1999, we did not have the small probe for transesophageal echocardiography and hence, in order to standardize our findings, we used the transthoracic data to assess all patients. We also noted that there was not much discrepancy of findings among the patients comparing who had transesophageal and who had transthoracic echocardiography after 1999. On follow-up, patients’ AR were quantified based on echocardiographic findings from the medical records. The AR gradient was quantified using pressure half-time measurement. Single measurement was taken for all the VSD. The size was taken as the intraoperative measurement size stated in the operative logbook by the surgeon.

Echocardiographic size of the VSD was not taken as with the presence of a prolapsed leaflet, the size measured might not be accurate. Other details about type of VSD, number of prolapsed cusps, type of AVr, cardiopulmonary bypass time, cross-clamp time, postoperative complications, and the need for reoperation were obtained from the operative notes and medical records. We define failure of the intervention if AR is more than moderate on pressure half-time measurement. We also tested all the valves with saline leak test intraoperatively and we accept success of the intervention if the three aortic cusps come together and hold the saline with not much leak into the ventricle.

### Statistical Analysis

All data were entered and analyzed using IBM Corp. Released 2011, IBM SPSS Statistics for Windows, Version 20.0, Armonk, NY: IBM Corp. Statistical significance was set at a *P*-value of < 0.05.

Preoperative AR grades were divided into three categories: mild, moderate, and severe AR. These categorical variables were compared with other variables using Pearson’s chi-squared test.

Differences in parametric variables among those three groups were compared by one-way analysis of variance (or ANOVA).

Univariate regression analysis was used to determine factors affecting the outcome of AVr. Multivariate analysis was then performed if there was presence of a predictive factor. Freedom from reoperation was analyzed by using Kaplan-Meier actuarial survival analysis. The log rank test was used for comparisons of Kaplan-Meier freedom from reoperation curves.

## RESULTS

### Patient Population

A total of 261 patients who met the inclusion criteria between January 1992 and December 2014 were included in the study. The mean age at surgery was 10.6±9.3 years, with 60.9% of the patients being male. There were 170 (65%) Malay patients, 56 (21.5%) Chinese patients, 12 (4.6%) Indian patients, and 23 (8.8%) other origin patients.

Most of our patients (50.6%) had perimembranous VSD, followed by doubly committed subaortic VSD (46%), and muscular VSD (3.4%). The mean VSD size was 1.2±0.6 cm (range: 0.4 - 3.0 cm). Among these 261 patients with AR in the setting of VSD, 164 (62.8%) had mild AR, 54 (20.7%) had moderate AR, and 43 (16.5%) had severe AR.

**Fig. 1:**
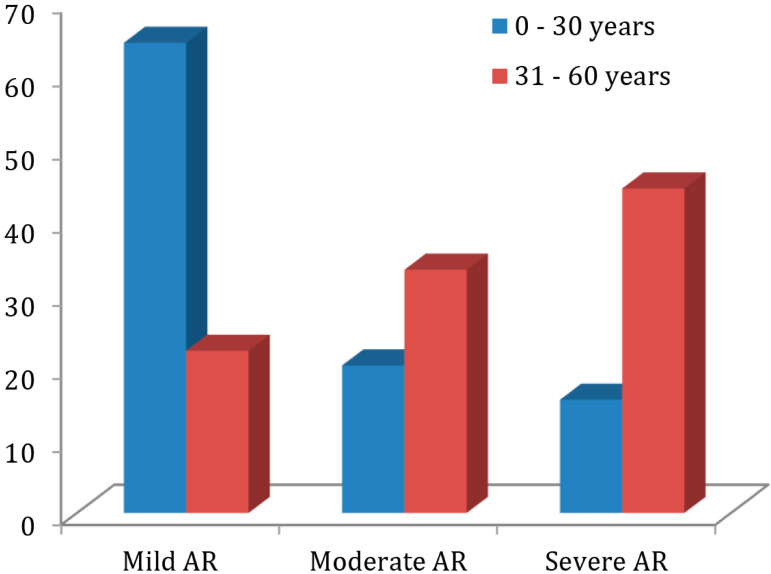
Grade of aortic regurgitation (AR) based on age group (P=0.022).

**Fig. 2:**
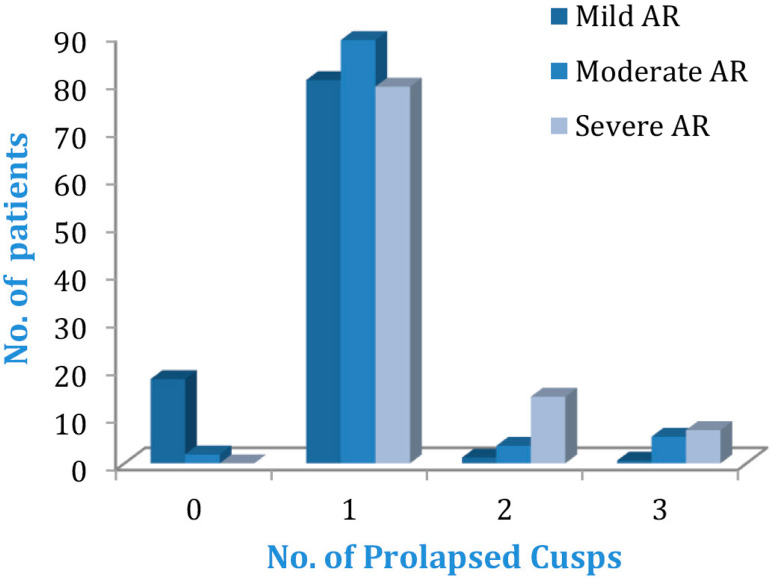
Number of prolapsed cusps and grade of aortic regurgitation (AR) (P<0.001).

Further cross tabulation (Pearson’s chi-squared test) revealed that there was significant association between VSD size and preoperative AR grade in this study (*P*=0.002). The preoperative AR grade was also significantly associated with the patients’ age (*P*=0.022) (Figure 1). Moreover, it was also found that there was a significant association between the number of prolapsed cusps and grade of AR preoperatively (*P*<0.001). Those patients with two or three prolapsed cusps had higher incidence of moderate and severe AR as compared to those with none or one prolapsed cusp, who had mainly mild AR (Figure 2).

### Operative Results

Aortic valve intervention for various grades of AR is shown in Table 1. AVr was performed in 84 patients (32.1%), AVR in 21 (8%), while the remaining 156 patients (59.8%) had NI performed for the aortic valve. Most of the patients with mild AR had NI done (90.9%), while the majority of those with moderate AR had AVr, which was 38 patients (77.8%). The other five (10%) patients had AVR, and six (12.8%) patients had NI in the moderate AR group. Forty-three patients had severe AR, 27 (62.8%) of them had AVr and 16 (37.2%) had AVR. In the mild AR group, 9.1% of the patients had valve repair, and all these patients underwent repair as the right coronary cusp was prolapsed into the VSD defect, and because of this excess tissue in the defect the aorta was also opened and the aortic valves were examined. The decision to repair was made by the operating surgeon and out of the 15 mild AR cases that were repaired, 12 had Trussler’s Repair and three had leaflet plication. On the contrary, there was 12.2% of moderate AR that was not intervened as the operating surgeon felt that there was adequate leaflet coaptation and there was no leaflet pathology to be addressed.

**Table 1:** Aortic valve intervention for the study population (N=261).

		Intervention			
		AVr	AVR	NI	Total
Grade of AR	Mild	15 (9.1%)	0 (0%)	149 (90.9%)	164 (100%)
	Moderate	42 (77.8%)	5 (9.2%)	7 (13.0%)	54 (100%)
	Severe	27 (62.8%)	16 (37.2%)	0 (0%)	43 (100%)
Total		84	21	156	261

AR=aortic regurgitation; AVr=aortic valve repair; AVR=aortic valve replacement; NI=no intervention

In this study, 84 patients underwent AVr, where 50 out of the 84 (59.5%) patients had Trussler’s repair, followed by commissural-related repair (15.5%), leaflet plication (13.1%), and others (11.9%). It was shown that there is a significant association between the type of repair performed and the number of prolapsed cusps in this study (*P*=0.002). Various factors were associated with the decision on the type of aortic valve intervention, especially when dealing with moderate and severe AR. It was shown in this study (Table 2), that the following factors had significant association with the type of aortic valve intervention (AVr/AVR/NI): age (*P*<0.001), size of VSD (*P*<0.001), grade of preoperative AR (*P*<0.001), and number of prolapsed cusps (*P*<0.001).

**Table 2:** Patients’ characteristics for various types of aortic valve intervention.

	AVr	AVR	NI	Total	*P*-value
Age (years)					
Range	1 – 53	9 – 59	0.3 – 51		0.001
Median	9	25	7		
**Race**					
Malay	52 (30.6%)	10 (5.9%)	108 (63.5%)	170	0.265
Indian	4 (33.3%)	3 (25%)	5 (41.7%)	12	
Chinese	20 (35.7%)	6 (10.7%)	30 (53.6%)	56	
Others	8 (34.8%)	2 (8.7%)	13 (56.5%)	23	
**Sex**					
Male	57 (35.8%)	12 (7.5%)	90 (56.7%)	159	0.286
Female	27 (26.5%)	9 (8.8%)	66 (64.7%)	102	
**Type of VSD**					
PM VSD	41 (31%)	10 (7.6%)	81 (61.4%)	132	0.903
DCSA VSD	39 (32.5%)	10 (8.3%)	71 (59.2%)	120	
Muscular VSD	4 (44.4%)	1 (11.2)	4 (44.4%)	9	
**Size of VSD (cm)**					
Mean	1.395	1.495	1.090		0.001
**Degree of AR**					
Mild AR	15 (9.1%)	0 (0%)	149 (90.9%)	164	0.001
Moderate AR	42 (77.8%)	5 (9.3%)	7 (13%)	54	
Severe AR	27 (62.8%)	16 (37.2%)	0 (0%)	43	
**Number of prolapsed cusps**					
0	0 (0%)	0 (0%)	30 (100%)	30	0.001
1	75 (35%)	13 (6.1%)	126 (58.9%)	214	
2	7 (70%)	3 (30%)	0 (0%)	10	
3	2 (28.6%)	5 (71.4%)	0 (0%)	7	
**Immediate postoperative period**					
Success	82 (97.6%)	21 (100%)	155 (99.4%)	258	0.423
Failure	2 (2.4%)	0 (0%)	1 (0.6%)	3	
**Follow-up**					
Success	63 (81.8%)	21 (100%)	142 (95.9%)	226	0.017
Failure	14 (18.2%)	0 (0%)	6 (4.1%)	20	
Missing data	7 (46.7%)	0 (0%)	8 (53.3%)	15	
**Mode of follow-up**					
Lost to follow-up	15 (68.2%)	1 (4.5%)	6 (27.3%)	22	0.001
Discharged	28 (38.4%)	9 (12.3%)	36 (49.3%)	73	
Still on follow-up	27 (23.7%)	8 (7.0%)	79 (69.3%)	114	
Transferred out	14 (26.9%)	3 (5.8%)	35 (67.3%)	52	

AR=aortic regurgitation; AVr=aortic valve repair; AVR=aortic valve replacement; DCSA=doubly committed subaortic; NI=no intervention; PM=perimembranous; VSD=ventricular septal defect

In terms of age, those of the older age group (≥ 31 years) had a higher percentage of AVR (55.6%), while those in the younger age group (≤ 30 years) had a higher percentage of NI (61.1%) and AVr (32.5%) (*P*<0.001). In this study, it was also noted that the type of aortic valve intervention (AVr/AVR/NI) was significantly affected by the number of prolapsed cusps (*P*<0.001). Most of patients with three prolapsed cusps had AVR (71.4%) done, while patients with none or one prolapsed cusp had NI done (100% and 58.9%, respectively).

In the immediate postoperative period, there was one failure of repair in the mild AR group and another failure in the moderate AR group, but no failure in the severe AR group (Table 3). There was also one failure in the mild AR group who did not have any intervention. During the follow-up period (Table 4), in the mild AR group, there were two failures in the AVr group and six failures in the NI group. The moderate AR group had seven failures in the AVr group, and the severe AR group had five failures in the AVr group. There were no failures in the immediate and follow-up periods for the patients who underwent AVR.

**Table 3:** Success of aortic valve intervention immediately after operation.

AR Grade		Postoperative success		Total
		Success	Failure	
Mild AR	AVr	14 (93.3%)	1 (6.7%)	15 (100%)
	AVR	-	-	-
	NI	148 (99.3%)	1 (0.7%)	149 (100%)
Moderate AR	AVr	41 (97.6%)	1 (2.4%)	42 (100%)
	AVR	5 (100%)	0 (0%)	5 (100%)
	NI	7 (100%)	0 (0%)	7 (100%)
Severe AR	AVr	27 (100%)	0 (0%)	27 (100%)
	AVR	16 (100%)	0 (0%)	16 (100%)
	NI	-	-	-

AR=aortic regurgitation; AVr=aortic valve repair; AVR=aortic valve replacement; NI=no intervention

**Table 4:** Success of aortic valve intervention during follow-up.

AR Grade		Success on follow-up		Total
		Success	Failure	
Mild AR	AVr	11 (84.6%)	2 (15.4%)	13 (100%)
	AVR	-	-	-
	NI	136 (95.8%)	6 (4.2%)	142 (100%)
Moderate AR	AVr	31 (81.6%)	7 (18.4%)	38 (100%)
	AVR	5 (100%)	0 (0%)	5 (100%)
	NI	6 (100%)	0 (0%)	6 (100%)
Severe AR	AVr	21 (80.8%)	5 (19.2%)	26 (100%)
	AVR	16 (100%)	0 (0%)	16 (100%)
	NI	-	-	-

AR=aortic regurgitation; AVr=aortic valve repair; AVR=aortic valve replacement; NI=no intervention

Univariate analysis was performed to determine the factors influencing the outcome of AVr on follow-up to improve patient selection in the future. Factors such as age, type of VSD, VSD size, preoperative grade of AR, cross-clamp time, cardiopulmonary bypass time, type of AVr, and AR grade immediately after operation, as well as the number of prolapsed cusps, were analyzed (Table 5). Age and type of AVr significantly affected the outcome of AVr (*P*<0.05). We found that younger patients and those who underwent leaflet plication as their repair technique had better outcome compared to others.

**Table 5:** Univariate analysis (Cox regression) for predictors of success of aortic valve repair on follow-up.

	Beta coefficient	HR (95% CI)	*P*-value
Age	0.039	1.040 (1.006 – 1.074)	0.020
Type of VSD	-0.066	0.936 (0.789 – 1.111)	0.451
VSD size	0.065	1.067 (0.699 – 1.627)	0.764
Preoperative AR grade	-0.135	0.873 (0.583 – 1.329)	0.512
Cross-clamp time	0.001	1.001 (0.991 – 1.010)	0.899
Bypass time	-0.004	0.996 (0.988 – 1.005)	0.396
Type of AVr	0.523	1.687 (1.054 – 2.699)	0.029
Postoperative AR grade	0.175	1.191 (0.846 – 1.678)	0.317
No. of prolapsed cusps	-0.213	0.808 (0.105 – 6.245)	0.838

AR=aortic regurgitation; AVr=aortic valve repair; CI=confidence interval; HR=hazard ratio; VSD=ventricular septal defect

Using similar factors, univariate analysis was also performed to determine the factors affecting reoperation (Table 6). It was noted that, in this study, the VSD size as well as the postoperative AR grade significantly affected reoperation (*P*-values of 0.048 and < 0.001, respectively).

**Table 6:** Univariate analysis (Cox regression) for predictors of reoperation in the aortic valve repair group.

	Beta coefficient	HR (95% CI)	*P*-value
Age	0.027	1.028 (0.956 – 1.105)	0.458
Type of VSD	0.064	1.066 (0.652 – 1.743)	0.798
VSD size	1.032	2.807 (1.008 – 7.815)	0.048
Preoperative AR grade	0.532	1.703 (0.569 – 5.094)	0.341
Cross-clamp time	-0.009	1.001 (0.991 – 1.010)	0.565
Bypass time	-0.006	0.992 (0.963 – 1.021)	0.624
Type of AVr	-0.285	0.752 (0.340 – 1.664)	0.482
Postoperative AR grade	3.039	20.89 (4.828 – 90.354)	<0.001
No. of prolapsed cusps	0.341	1.406 (0.415 – 4.756)	0.584

AR=aortic regurgitation; AVr=aortic valve repair; CI=confidence interval; HR=hazard ratio; VSD=ventricular septal defect

A residual moderate or severe AR in the immediate postoperative period has been shown to be the main reason for reoperation in our cohort of patients. Pertaining to VSD size, we also found that the patients with smaller size VSD had higher incidence for reoperation.

There was no hospital mortality among these 261 patients. Overall freedom from reoperation at 10 and 15 years were 86.9% and 85.6%, respectively, for AVr, and freedom from reoperation for AVR at 10 and 15 years were 88.9% and 88.9%, respectively.

**Fig. 3:**
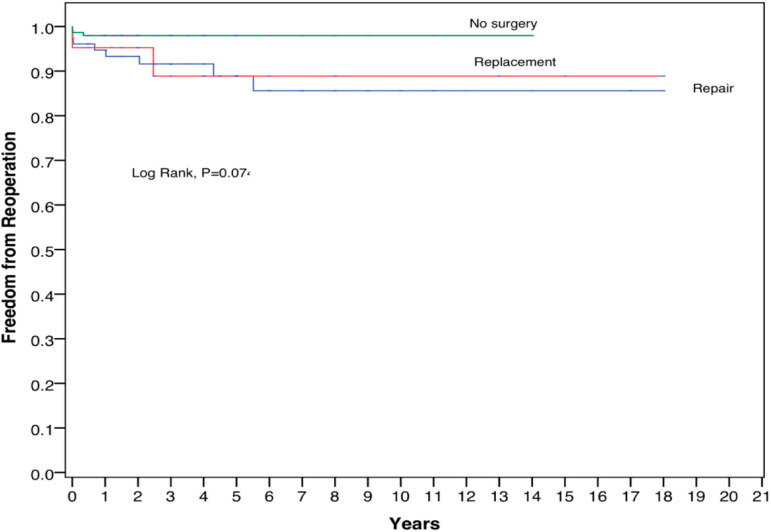
Kaplan-Meier curve – freedom from reoperation for various aortic valve interventions. Patient numbers according to the intervention done: no surgery, 156 patients; aortic valve replacement, 21 patients; and aortic valve repair, 84 patients.

**Fig. 4:**
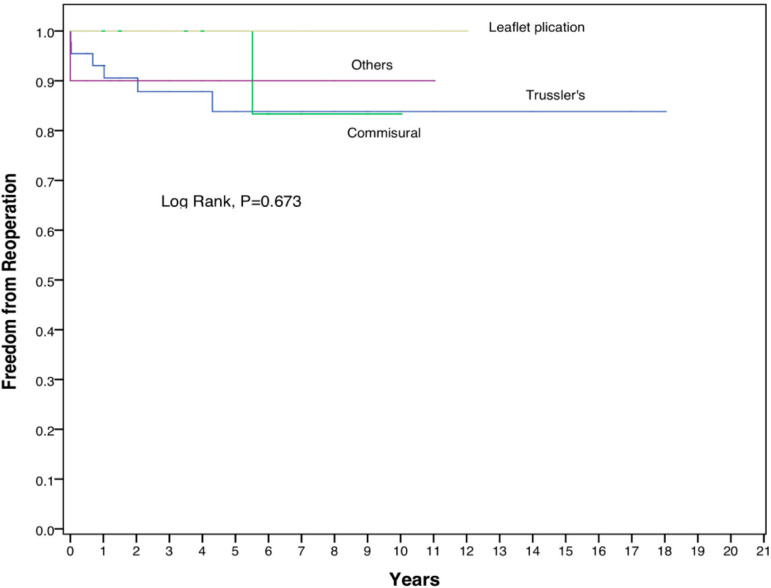
Kaplan-Meier curve – freedom from reoperation for various methods of aortic valve repair. Patient numbers based on the type of aortic valve repair: Trussler’s repair, 50 patients; commissural-related repair, 13 patients; leaflet plication, 11 patients; others, 10 patients.

Among those with NI, freedom from reoperation at 10 and 15 years were 98.0% for both periods. The Kaplan-Meier curve in Figure 3 shows that there are no significant differences in the freedom from reoperation among all three groups (log rank, *P*-value of 0.074). Figure 4 depicts the freedom from reoperation for various methods of AVr. There is no significant difference in the freedom from reoperation among all four repair groups (*P*-value of 0.673). For those who underwent leaflet plication as a method of AVr, freedom from reoperation at 10 and 15 years were 100% in this study. For Trussler’s repair, the freedom from reoperation was 83.8% at 10 years as well as 15 years, while for commissural-related procedures, the freedom from reoperation at 10 and 15 years were 84% and 83.3%, respectively.

All patients were followed up for a mean time of 13.9±3.5 years (range: 0.5 - 25 years). Among the 261 patients who were included in this study, 114 (43.7%) of them are still under follow-up, 73 (28%) have been discharged from follow-up, while 52 patients (19.9%) have been transferred to another state hospital for follow-up and 22 (8.4%) patients are lost to follow-up.

The patients with moderate and severe AR had longer follow-up (mean time of 15.6±4.6 years and 15.4±4.2 years, respectively). The follow-up duration was shorter in the mild AR group (mean time of 2.9±2.3 years).

## DISCUSSION

This study demonstrates that patients with VSD and concomitant AR have several treatment options. Addressing the aortic valve at an earlier age has been shown to provide a better outcome^[1]^.

This is due to the fact that, if left untreated, AR will progress to a more severe form in the older age, necessitating AVR rather than AVr^[2,3]^.

In most patients, AR tends to be detected between the ages of three to eight years^[4]^. This is consistent in our study, in which the median age at operation was eight years. Older patients presented with more severe AR (*P*=0.022). The median age for the AVR group was older (25 years old) while those who underwent AVr were younger (median age of nine years).

Previous reports^[2,3]^ have pointed out that the grade of AR is associated with the size of VSD, and this relationship was also seen in this study (*P*-value of 0.002). A large VSD has been shown to have a lower AR gradient. The reason for this has been mainly related to the venturi effect that happens to the leaflets. In a smaller VSD defect, the suction effect to the leaflet is far greater than in a larger defect, and this displace the cusp even more and exaggerates the prolapse of the leaflets, hence worsening the AR^[2]^. We had 15 patients with VSD size of > 2.1 cm; 40% of these patients had mild AR, 33% had moderate AR, while only 27% had severe AR. This showed that the larger the VSD size, the lesser the severity of AR. In a small VSD, the venturi effect is far greater than in larger VSDs. This also will exert more strain to the affected leaflet.

Constant pulling effect on the affected leaflet can damage the microarchitecture of the leaflet and this can predispose the leaflet to fail in coming years.

Echocardiography is not very reliable in providing the true size of the VSD, especially in the presence of AR, because the true size tends to be underestimated^[5]^. The best method would still be intraoperative measurement of the VSD defect, which was performed in this study.

Although the syndrome of VSD and AR has been long recognized as a clinical entity, there is still no common agreement about its management, particularly with regards to the indication, timing, and technical details of the surgical procedure^[6,7]^. The ideal management should be one which is safe, simple, reproducible, and durable, and should deal with all the anatomic components of the syndrome, preferably at a young age. In practice, achieving these deceptively simple goals is difficult.

In our series, all patients with a preoperative moderate or severe AR gradient had their aortic valves inspected and assessed during surgery. We had a few criteria which we used to assess the valve on table and this included the effective height of each leaflet, the support from the commissures, symmetrical apposition of the leaflet by using the Nodule of Arientus as a guide, and leaflet appearance. Leaflets which have rolled edges, elongated and asymmetrical, underwent repair even if the AR was only mild. We also had patients with had moderate AR, but with normal cusp and commissures, who had NI to their aortic valve. All this assessment was solely decided by the operating surgeon and this is one of the main limitations of our study as we had no standard criteria to select patients for AVr.

In the mild AR group, we had two AVr that failed, both of these using the Trussler’s technique, and we had six patients that had NI in the mild group progressing to more than moderate AR after the VSD was closed. We feel that the Trussler’s repair has its limitations, the patient must be carefully selected, and it is not suitable if there is leaflet elongation. The six patients who had NI to their valve were found to be older children (range: 6 - 8 years old) and all of them had a perimembranous VSD with a size < 1 cm. The moderate AR group had six patients who had NI, and these patients still had a competent aortic valve on their latest follow-up. We feel that as long the leaflet has no major structural abnormality and it has good commissural support, the closure of the VSD itself should be sufficient. This, however, must be an accurate assessment by the operating surgeon.

Previous studies^[2,11]^ have shown associations between preoperative AR grade and subsequent AR progression as well as the type of intervention. In this study, we further clarify that such association was present between the preoperative AR grade and the progression of the AR in coming years (*P*-value of < 0.001). In addition to the preoperative AR grade, older age at VSD repair (*P*<0.001) as well as VSD size (*P*<0.001) also predicted the type of aortic valve intervention. We had 15 patients with mild AR that underwent AVr and this was mainly due to the presence of right coronary cusp prolapse in the doubly committed subarterial group of patients. Although the AR was mild, we decided to plicate the leaflet as it was elongated into the VSD defect as a windsock deformity.

In our study, the median age for AVR was 25 years and none of our patients who needed AVR was younger than nine years old. If the VSD shunt persisted and patients grew older, prolapse would get worse with time and even progressed to sinus of Valsalva aneurysm. The more the damage to the aortic valve, the more likely AVR will be needed^[3]^. This may be the reason why older age at VSD repair predicts the necessity for AVR.

Moreover, it has also been reported that the longer the wait for surgery, the more morphological changes happen to the aortic cusps, making AVr less suitable^[8]^.

AR in patients with VSD is commonly associated with prolapse of the aortic valve cusps, with elongation of the free edge^[9]^. Therefore, attempts to surgically treat AR are directed towards AVr.

In this study, the number of prolapsed aortic cusps were significantly associated with the preoperative grade of AR (*P*=0.001), and therefore, it had a significant association with the type of aortic valve intervention (*P*<0.001). Besides that, there was also significant association between the type of AVr performed with the number of prolapsed cusps in our study (*P*=0.002).

Indications for AVr have not been explicitly defined in previous studies. Furthermore, previous reports on the long-term outcome of VSD repair with concomitant AVr were based on a relatively small number of patients.

Among the 84 patients who had AVr done, most of them underwent Trussler’s repair (59.5%). The type of AVr employed was significantly associated with the number of prolapsed cusps (*P*<0.001). Those with three cusps prolapsed mainly had an AVR done (71.4%). Replacement of an entire aortic valve is not done when only one cusp is prolapsed^[10]^.

Therefore, in those patients with none or one prolapsed cusp, AVr was performed instead of AVR.

Overall, our 10- and 15-year freedom from reoperation for AVr were 88.9% and 82.6%, respectively. This compares favorably with other published series which estimates range from 85% at 10 years to 64% at 15 years^[5,11]^.

It is important to identify the possible risk factors that are associated with failure of AVr so that better patient selection can be practiced, hoping for better outcome in the future. In this way, the freedom from reoperation for AVr will be acceptable, hence reducing the necessity for AVR.

The advantages (growth potential, avoidance of anticoagulation, and minimal thromboembolic risk) and disadvantages of AVr (residual lesions and need for later valve surgery) must be balanced against the outcomes of AVR so that AVr becomes an attractive and justifiable alternative^[4]^.

It was also noted in this study that the VSD size as well as the postoperative AR grade significantly affected reoperation (*P*-value of 0.048 and < 0.001, respectively).

There was no hospital mortality noted in this study period consistent with most other published studies^[5,12]^.

This was a retrospective study through a long period. Indications for surgical intervention on the aortic valve have changed, as have AVR options, techniques of repair, and available material. We did not have a large enough group of patients for AVr as well as AVR, which may have affected our outcome. Although we could not identify a significant difference in freedom from reoperation between AVr and AVR, the curves do diverge. It is quite possible that if we had had more patients, this divergence would have reached significance.

AVr is an effective and durable technique for the surgical treatment of AR in patients with VSD. In this study, AVr achieved a satisfactory success rate.

This stressed the importance of early detection and intervention prior to progression of AR, thus reducing the rate of AVR. However, longer follow-up with larger number of patients would provide a better assessment of the outcome of AVr in patients with syndrome of VSD and AR.

Over the years it has always been a debate to decide on when is it safe to intervene this group of patients. A recent publication^[13]^ highlighted that the youngest patient they operated on was at three months old, and this was also the same in our series. In that series, they also found that patients did better when they were operated at a younger age. A similar finding that we also found in our series. We, however, had a longer follow-up and patient cohort to support this finding in comparison with the recent study^[13]^.

The risk of cardiopulmonary bypass has always been the concern for young infants. There have been recent reports on transcatheter closure of VSD in aortic valve prolapse and AR^[14]^. The procedure has been shown to be safe and feasible in the setting of AR. In a recent review for the safety of device closure for VSD, it was found that percutaneous and perventricular device closure for VSD are safe and did not have any extra morbidity^[15]^.

The avoidance of cardiopulmonary bypass will further bring down the operative age and further protect the structural damage to the aortic valve. This will enable early VSD closure and probably NI for the aortic valve in coming years.

### Limitations

This is a retrospective study and most of the data were collected from the operative notes and patients' medical records. The decision to intervene the valve was solely of the operating surgeon and there was no strict criteria or guideline that was used as a guide for the valve intervention. The actual size of the VSD was also a size that was estimated by the operating surgeon.

## CONCLUSION

We can conclude from our study that all moderate and severe AR with small VSD in older patients with more than one cusp prolapse will need intervention to their aortic valve during the closure of the VSD.

**Table:** 

Authors' roles & responsibilities	
SK	Substantial contributions to the conception or design of the work; or the acquisition, analysis, or interpretation of data for the work; drafting the work or revising it critically for important intellectual content; agreement to be accountable for all aspects of the work in ensuring that questions related to the accuracy or integrity of any part of the work are appropriately investigated and resolved; final approval of the version to be published
SS	Substantial contributions to the conception or design of the work; or the acquisition, analysis, or interpretation of data for the work; drafting the work or revising it critically for important intellectual content; final approval of the version to be published
JD	Substantial contributions to the conception or design of the work; or the acquisition, analysis, or interpretation of data for the work; drafting the work or revising it critically for important intellectual content; final approval of the version to be published
RARM	Agreement to be accountable for all aspects of the work in ensuring that questions related to the accuracy or integrity of any part of the work are appropriately investigated and resolved; final approval of the version to be published
AY	Agreement to be accountable for all aspects of the work in ensuring that questions related to the accuracy or integrity of any part of the work are appropriately investigated and resolved; final approval of the version to be published
RS	Agreement to be accountable for all aspects of the work in ensuring that questions related to the accuracy or integrity of any part of the work are appropriately investigated and resolved; final approval of the version to be published
